# Supplementary material for: IL-33 facilitates rapid expulsion of the parasitic nematode Strongyloides ratti from the intestine via ILC2- and IL-9-driven mast cell activation
Source: PLoS Pathog. 2020 Dec 22;16(12):e1009121. doi: 10.1371/journal.ppat.1009121 (PMC7787685; doi:10.1371/journal.ppat.1009121)
Supplement: S1 Fig — IL-33 release by tissue explants BALB/c mice were left naïve (open circles) or s.c. infected with 2000 S. ratti L3 (closed circles). Mice were sacrificed day 2 and day 6 p.i. and lungs and small intestine prepared. Tissue explants corresponding to half a lung (ca 100 mg) or one tenth of the small intestine (ca 150 mg) were weighed, placed in 48 well plates in 250 μl serum-free RPMI 1640 medium supplemented with 100 U/ml Penicillin/Streptavidin and cOmplete Protease Inhibitor cocktail (Roche) and incubated for 24 h at 37°C. IL-33 in the SN was quantified using an IL-33 ELISA Kit (Invitrogen by Thermfisher Scientific) according to the manufacturer`s recommendation and normalized to the weight of the explants. Graphs show the combined results of 1 (day 2) or 3 independent experiments (n = 2–8 per group and experiment). Each symbol represents an individual mouse, bars show the mean, number indicate the p value and asterisk indicate statistically significant differences between groups (Mann-Whitney test). (PDF) [file ppat.1009121.s001.pdf]

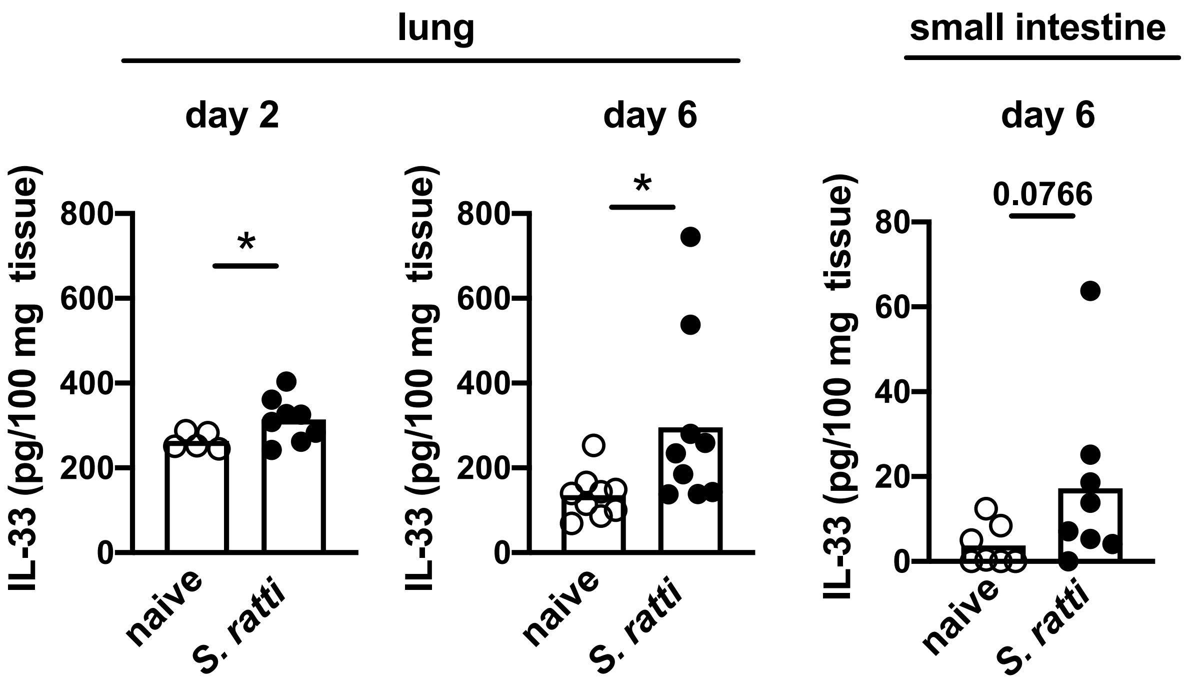

S1 Fig (related to Fig 1): IL-33 release by tissue explants.

BALB/c mice were left naïve (open circles) or s.c. infected with 2000 *S. ratti* L3 (closed circles). Mice were sacrificed day 2 and day 6 p.i. and lungs and small intestine prepared. Tissue explants corresponding to half a lung (ca 100 mg) or one tenth of the small intestine (ca 150 mg) were weighed, placed in 48 well plates in 250 µl serum-free RPMI 1640 medium supplemented with 100 U/ml Penicillin/Streptavidin and cOmplete Protease Inhibitor cocktail (Roche) and incubated for 24 h at 37°C. IL-33 in the SN was quantified using an IL-33 ELISA Kit (Invitrogen by Thermo Fisher Scientific) according to the manufacturer`s recommendation and normalized to the weight of the explants. Graphs show the combined results of 1 (day 2) or 3 independent experiments (n = 2-8 per group and experiment). Each symbol represents an individual mouse, bars show the mean, number indicate the p value and asterisk indicate statistically significant differences between groups (Mann-Whitney test).
