# Supplementary material for: IL-33 facilitates rapid expulsion of the parasitic nematode Strongyloides ratti from the intestine via ILC2- and IL-9-driven mast cell activation
Source: PLoS Pathog. 2020 Dec 22;16(12):e1009121. doi: 10.1371/journal.ppat.1009121 (PMC7787685; doi:10.1371/journal.ppat.1009121)
Supplement: S2 Fig — Intranasal application of IL-33 results in systemic elevation of IL-33 concentration and mucosal mast cell activation (A) Experimental procedure: BALB/c mice were treated i.n. (open circles) or i.p. (closed circles) with 1 μg rec. IL-33 3 h before and 24 h post S. ratti infection. Serum samples were taken at the indicated time points and (B) IL-33 and (C) mMCPT-1 concentration in the sera were quantified pre-treatment (0), 3 h, 1 and 3 days after treatment by ELISA. Shown are combined results from 2 independent experiments (n = 3–5; pre-treatment n = 2 per experiment and group) each symbol represents an individual mouse, bars show the mean and asterisk indicate statistically significant difference of the means compared to pre-treatment (one-way ANOVA). (PDF) [file ppat.1009121.s002.pdf]

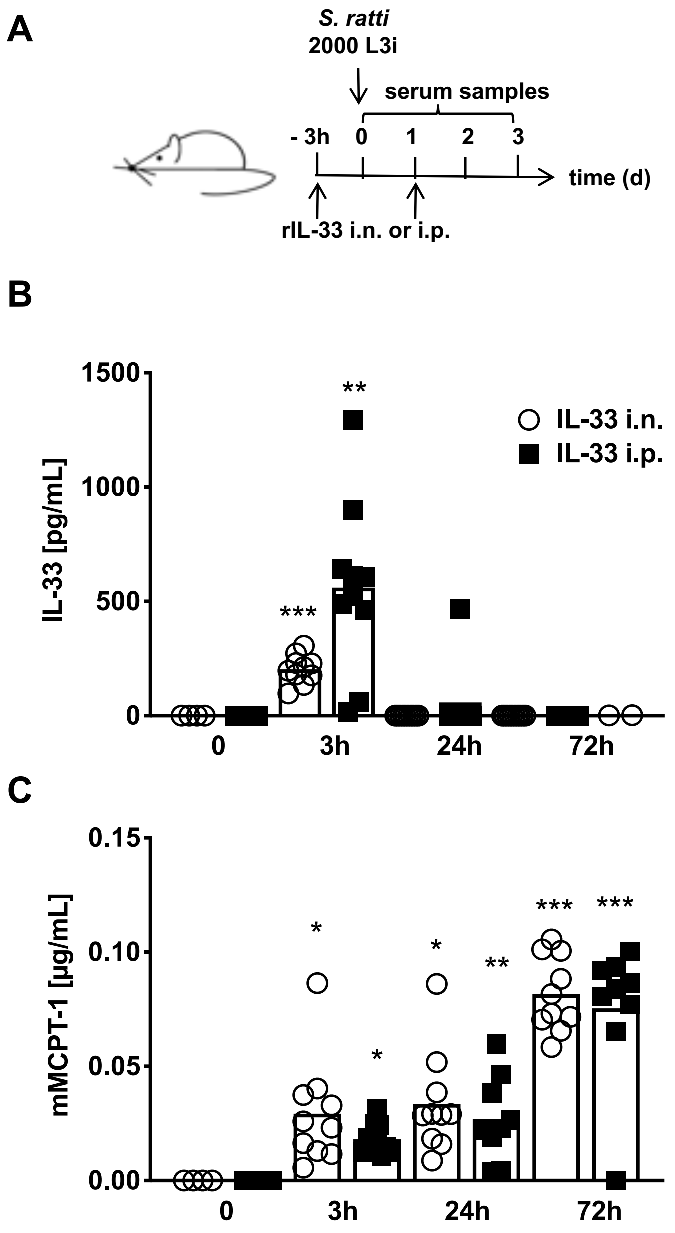

S2 Fig (related to Fig 2): Intranasal application of IL-33 results in systemic elevation of IL-33 concentration and mucosal mast cell activation

(A) Experimental procedure: BALB/c mice were treated i.n (open circles) or i.p. (closed circles) with 1 µg rec. IL-33 3 h before and 24 h post *S. ratti* infection. Serum samples were taken at the indicated time points and **(B)** IL-33 and **(C)** mMCPT-1 concentration in the sera were quantified pre-treatment (0), 3 h, 1 and 3 days after treatment by ELISA. Shown are combined results from 2 independent experiments (n = 3-5; pre-treatment n = 2 per experiment and group) each symbol represents an individual mouse, bars show the mean and asterisk indicate statistically significant difference of the means compared to pre-treatment (one-way ANOVA).
