# Supplementary material for: IL-33 facilitates rapid expulsion of the parasitic nematode Strongyloides ratti from the intestine via ILC2- and IL-9-driven mast cell activation
Source: PLoS Pathog. 2020 Dec 22;16(12):e1009121. doi: 10.1371/journal.ppat.1009121 (PMC7787685; doi:10.1371/journal.ppat.1009121)
Supplement: S3 Fig — Depletion of Gr-1+ cells (A) Experimental procedure: BALB/c mice received i.p. 350μg anti-Gr-1 mAb (clone RB6-8C5, squares) or isotype control (circles) one day before and one day after S. ratti infection. Mice were additionally treated with 1 μg of IL-33 (closed symbols) or with PBS (open symbols) 3 h before and 24 h post S. ratti infection. Frequency of Gr-1+ CD11b+ cells in the leukocyte gate of PBS were measured by flow cytometry at day 1 p.i. To this end cells were stained with anti-mouse/human CD11b-PerCP-Cy5.5 (M1/70) and anti-mouse Gr-1-BV421 (RB6-8C5) (both BioLegend, Germany), measured with an LSRII Cytometer (BD, Germany) and analyzed by FlowJo software. (B) Representative dot blots and (C) combined results of 2 independent experiments (n≥ ≥ 4 per experiment and group) showing frequency of granulocytes within PBL-leukocytes of the indicated groups are shown. Each symbol represents an individual mouse, bars represent the mean and asterisk indicate statistically significant differences of indicated groups (Kruskal-Wallis test with Dunn`s post test). (PDF) [file ppat.1009121.s003.pdf]

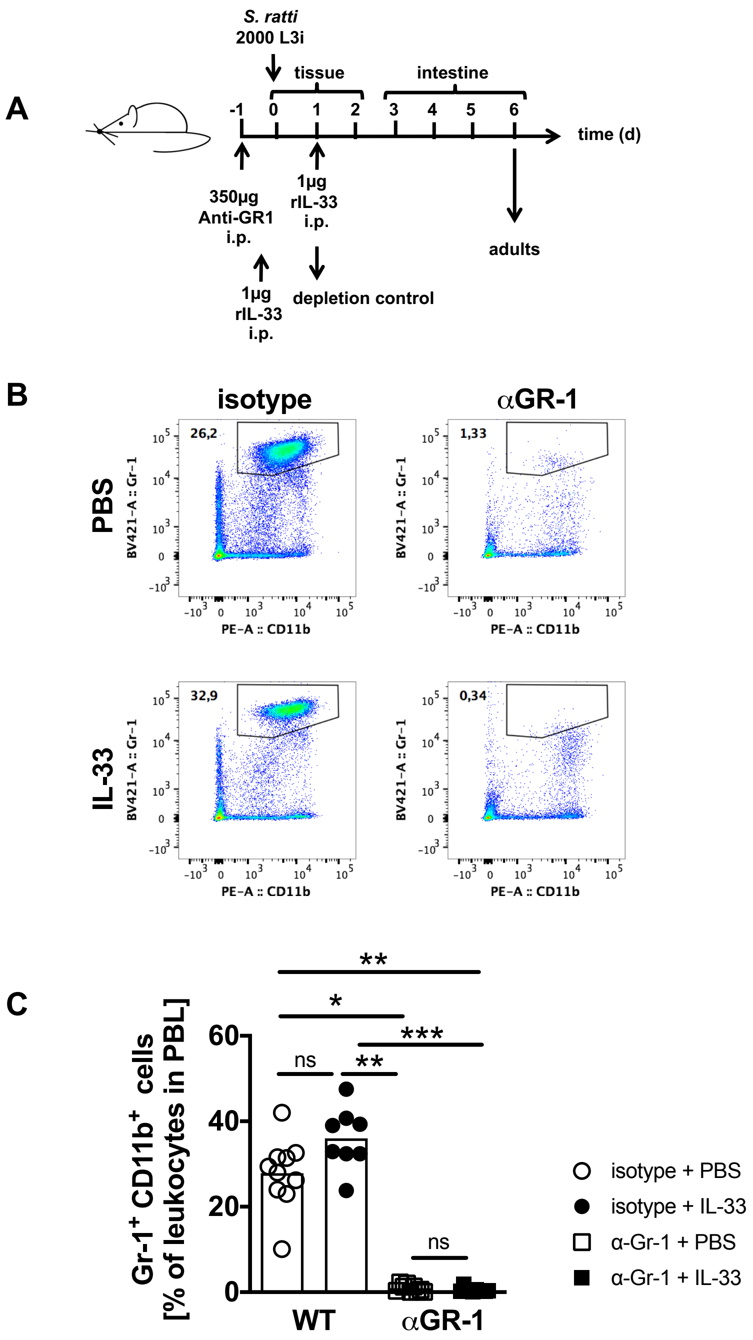

S3 Fig (related to Fig 4): Depletion of Gr-1$^+$ cells

(A) Experimental procedure: BALB/c mice received i.p. 350µg anti-Gr-1 mAb (clone RB6-8C5, squares) or isotype control (circles) one day before and one day after *S. ratti* infection. Mice were additionally treated with 1 µg of IL-33 (closed symbols) or with PBS (open symbols) 3h before and 24 h post *S. ratti* infection. Frequency of Gr-1$^+$CD11b$^+$ cells in the leukocyte gate of PBS were measured by flow cytometry at day 1 p.i. To this end cells were stained with anti-mouse/human CD11b-PerCp-Cy5.5 (M1/70) and anti-mouse Gr-1-BV421 (RB6-8C5) (both BioLegend, Germany), measured with an LSRII Cytometer (BD, Germany) and analyzed by FlowJo software. **(B)** Representative dot plots and **(C)** combined results of 2 independent experiments (n ≥ 4 per experiment and group) showing frequency of granulocytes within PBL-leukocytes of the indicated groups are shown. Each symbol represents an individual mouse, bars represent the mean and asterisk indicate statistically significant differences of indicated groups (Kruskal-Wallis test with Dunn`s post test).
