# Supplementary material for: IL-33 facilitates rapid expulsion of the parasitic nematode Strongyloides ratti from the intestine via ILC2- and IL-9-driven mast cell activation
Source: PLoS Pathog. 2020 Dec 22;16(12):e1009121. doi: 10.1371/journal.ppat.1009121 (PMC7787685; doi:10.1371/journal.ppat.1009121)
Supplement: S4 Fig — Gating of ILC2 ILC2 gating strategy is displayed for splenic cells isolated from a BALB/c RAG-/- mouse treated with 1 μg rec. IL-33. Cells were stained for 25 minutes at 4°C with Biotin-labeled lineage cocktail (targeting mouse CD11b, CD8, CD19, CD11c, CD3, TCRβ, TCRγδ, Gr-1, CD5, CD49b, TER-119 and NK1.1) and PE-Cy7-labeled anti-mouse CD90.2 antibody and BV421-labeled anti-mouse CD127 antibody. Subsequently, cells were washed and stained for 15 minutes at 4°C with PerCP Cy5.5-labeled Streptavidin. For intracellular staining, first cells were fixed and permeabilized using the Thermofisher Scientific Foxp3/Transcription factor staining buffer set according to the manufacturer’s protocol. Intracellular staining was performed using the following antibodies: AF488-labeled anti-mouse GATA3 antibody, PE-labelled anti-mouse Eomes antibody, APC-labeled anti-mouse RorγT antibody, and PE/Dazzle594-labeled anti-mouse T-bet antibody. Cells were measured using an LSRII Cytometer (BD, Germany) and analyzed by FlowJo software. (PDF) [file ppat.1009121.s004.pdf]

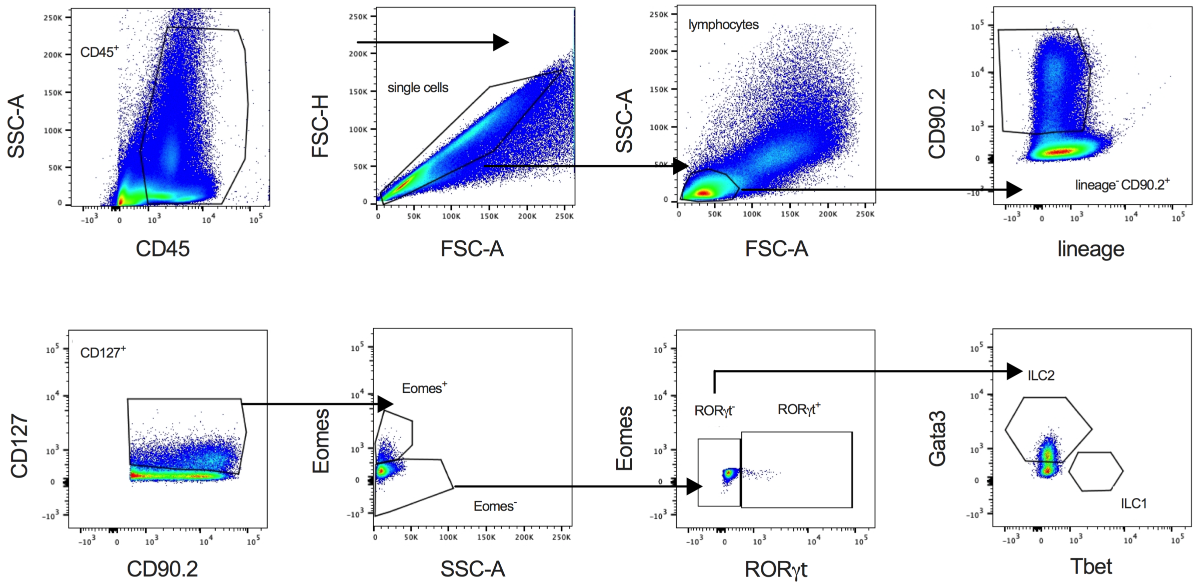

S4 Fig (related to Fig 5): Gating of ILC2

(A) ILC2 Gating strategy is displayed for splenic cells isolated from a BALB/c RAG$^{-/-}$ mouse treated with 1 µg rec. IL-33. Cells were stained for 25 minutes at 4°C with Biotin-labelled lineage cocktail (targeting mouse CD11b, CD8, CD19, CD11c, CD3, TCRβ, TCRγδ, Gr-1, CD5, CD49b, TER-199 and NK1.1) and PeCy7-labeled anti-mouse CD90.2 antibody and BV421-labeled anti-mouse CD127 antibody. Subsequently, cells were washed and stained for 15 minutes at 4°C with the secondary PerCP Cy5.5-labeled Streptavidin. For intracellular staining, first cells were fixed and permeabilized using the Thermofisher Scientific Foxp3/Transcription factor staining buffer set according to the manufacturer's protocol. Intracellular staining was performed using the following antibodies: AF488-labeled anti-mouse GATA3 antibody, PE-labelled anti-mouse Eomes antibody, APC-labelled anti-mouse RorγT antibody, and PE/Dazzle594-labeled anti-mouse T-bet antibody. Cells were measured using an LSRII Cytometer (BD, Germany) and analyzed by FlowJo software.
