# Supplementary material for: IL-33 facilitates rapid expulsion of the parasitic nematode Strongyloides ratti from the intestine via ILC2- and IL-9-driven mast cell activation
Source: PLoS Pathog. 2020 Dec 22;16(12):e1009121. doi: 10.1371/journal.ppat.1009121 (PMC7787685; doi:10.1371/journal.ppat.1009121)
Supplement: Attachment — Submitted filename: response to reviewers.pdf [file ppat.1009121.s013.pdf]

Dear Drs Nair, Loke, Haldar and Malim,

Thank you very much for rapid and expert revision of our manuscript. We would also like to thank the reviewers for devoting their time. Here we submit a revised version that takes into account their comments and critique. In summary, we performed a quantification of endogenous IL-33 that is released during *S. ratti* infection in lung and small intestine (new Supplementary Figure S1). To further explore the significance of endogenous IL-33, we now have evaluated mast cell responses in mice where we suppressed (HpARI) or stabilized (CCP1/2) endogenous IL-33 responses during infection. These results are shown in the new Figure 3B-C and demonstrate that endogenous IL-33 promotes mast cell activation. Thus, endogenous IL-33 promotes intestinal anti-*S. ratti* immunity via the very same key mechanism that we described for the treatment with exogenous recombinant IL-33. Below we respond point-by-point with the original comments in blue.

Editor's comments:
Please address requests from Reviewers 1 and 2, specifically to provide data and results that further explore the significance of endogenous IL-33, and evaluate mast cell responses. Please also address Reviewers minor revision requests.

Reviewer's Responses to Questions

Part I - Summary
Please use this section to discuss strengths/weaknesses of study, novelty/significance, general execution and scholarship.

Reviewer #1: The authors have done a detailed study of the role of administered IL-33 on the primary response of BALB/c mice to the pathogen S. ratti. Studies focused on the role of endogenously produced IL-33 were more limited, as shown in some of the data in Fig. 1. As the authors state in the abstract: "Blockade of endogenous IL-33 using a helminth-derived IL-33 inhibitor elevated intestinal parasite burdens while stabilization of endogenous IL-33 or application of recombinant IL-33 reciprocally reduced intestinal parasite burdens." In all of the remaining figures, the approach was to treat BALB/c mice, or mutants on the BALB/c background, with exogenous IL-33, and to analyze the dependence of an appropriate response to IL-33 on various components of the primary immune response to S. ratti.As a result, except for some of the data in Fig. 1, all studies are pharmacological analyses of the consequences of administered IL-33 on the BALB/c mouse response to S. ratti infection. The results of such studies show what the amounts of exogenous IL-33 used in the experiments can do, and the dependence on such results on particular immune cells. However, whether endogenous IL-33, in the amounts that can be produced in vivo, is able to mediate the same effects apparently has not yet been shown.The following changes will render this report more useful in the growing literature regarding the primary immune response to S. ratti.

1) The authors should more clearly describe which experiments pertain to endogenous IL-33 (some of the data in Fig. 1) and which experiments use administered exogenous IL-33. They also should note explicitly that the latter experiments provide evidence of what endogenous IL-33 might do in this response, but do not prove that production of endogenous IL-33 has all of the effects shown for administered exogenous IL-33. More work addressing the role of endogenous IL-33 in this response would greatly increase the relevance of the study.

In response to this valid point, we have demonstrated release of IL-33 during *S. ratti* infection (new Supplementary Figure S1 and page 5, lines 4 ff). We also pointed out the difference between application of recombinant IL-33 and suppression or stabilization of endogenous IL-33 throughout the manuscript including the abstract. It is true that most of the mechanistic studies in our manuscript were performed using application of exogenous IL-33. Nevertheless, we argue that using inhibitors (HpARI) and enhancers (CCP1/2) to IL-33 is directly addressing the function of endogenous IL-33 in control of intestinal parasite burden as shown in Figure 1. To provide further mechanistic studies addressing the function of endogenous IL-33 we have now evaluated mast cell responses in mice where we suppressed (HpARI) or stabilized (CCP1/2) endogenous IL-33, these additional results are shown in the new Figure 3B-C (page 7, lines 16 ff). These data clearly demonstrate that endogenous IL-33 promotes mast cell activation which is the key mechanism of IL-33-induced intestinal anti-*S. ratti* immunity described for the treatment with exogenous IL-33. As regards the follow-up experiments assessing the effect of exogenous rec. IL-33 administration on ILC-, mast cell- and IL-9R-deficient mice, we did not repeat these all using HpARI or CCP1/2 administration. However, we believe that our data is strongly supportive of the hypothesis that the IL-33 pathway is critical in the mast cell response to the parasite, and that our approach of exogenous IL-33 administration allowed us to more sensitively dissect out the immunological mechanisms at play. We have included this reasoning in the revised manuscript (page 13, lines 6 ff).

2) Since the results in Fig. 1 are the only data which focus on endogenous IL-33, more information about the natural

products analyzed (i.e., the full length helminth-derived IL-33 inhibitor and the truncated version of that) should be provided. Specifically, how clear is the evidence that the effects of these agents, given in the amounts used in the authors' experiments, are solely attributable to effects on IL-33?

In previous publications we have shown that HpARI is a highly-specific binder of IL-33. For instance, no binding to the closely related cytokine IL-1α, or the functionally similar nuclear cytokine HMGB1 were observed (1). Furthermore, while HpARI shows potent suppression of IL-33-mediated activation of ILC2s, it cannot suppress IL-25-mediated ILC2 activation – see attached Figure 1). Likewise, as CCP1/2 is a truncation of HpARI, its effects are restricted to IL-33. In vivo, CCP1/2 can amplify responses to exogenous IL-33, or endogenous IL-33 induced in highly IL-33-dependent Alternaria allergen administration or *N. brasiliensis* infection (2). We have included these information in the revised version of the manuscript (page 10, lines 3 ff and page 13, lines 6 ff). We have also added the details describing the production and purification of these proteins in the materials and methods section.

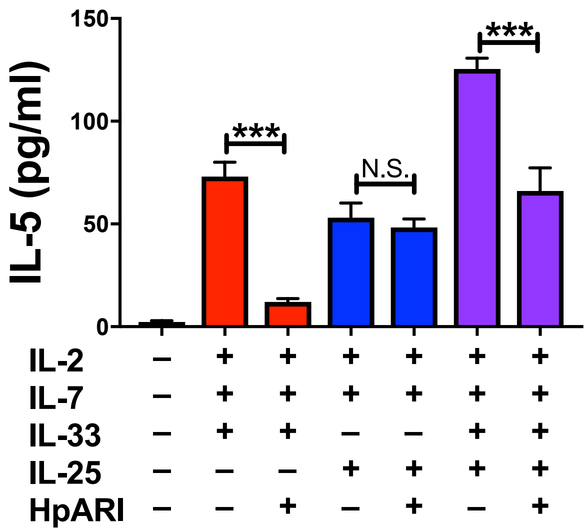

Figure 1: C57BL/6 bone marrow cells were stimulated with the cytokine cocktails indicated (all cytokines at 10 ng/ml) for 5 days in the presence or absence of 1 ug/ml HpARI. IL-5 was measured in supernatants by ELISA.

3) The authors should note more explicitly that their results pertain to the response to S. ratti in BALB/c mice, and such results may differ among other strains of mice.
This is an excellent point since we have observed strain-specific differences in immune regulation during S. ratti infection ((3) and unpublished observations). Although we did not analyze C57BL/6 mice so far, the function of IL-33 during *S. venezuelensis* infection was described by Yasuda and coworkers (4) using IL-33-deficient mice on C57BL/6 background. We have added this reasoning in the discussion. (page 11, lines 24 ff).

Minor points:
1. The attached version of the manuscript has noted minor typographical problems, which need to be addressed.
We performed an additional round of proof-reading.

Reviewer #2: This elegant study identifies that boosting IL-33 promotes ILC2 IL-9 driven mast cell responses to promote resolution of S.ratti infection.
This is a novel for identifying the role of IL-33 driving mast cell responses in this model infection. Observations are robustly tested using a range of appropriate transgenic mouse strains.

Reviewer #3: This manuscript presents a series of very well-designed experiments that demonstrate that IL-33 triggers rapid mast cell-mediated expulsion of Strongyloides ratti from the intestine, independent of the adaptive immune system, basophils, eosinophils but dependent on IL-9 receptor and innate lymphoid cells. The paper is very clearly written and significantly advances our knowledge of host parasite-relationships and intestinal immune mechanisms.

We thank the reviewers for their supportive comments. Specific comments are responded to below.

Part II – Major Issues: Key Experiments Required for Acceptance
Please use this section to detail the key new experiments or modifications of existing experiments that should be absolutely required to validate study conclusions.

Generally, there should be no more than 3 such required experiments or major modifications for a "Major Revision" recommendation. If more than 3 experiments are necessary to validate the study conclusions, then you are encouraged to recommend "Reject".

Reviewer #1: 1) The authors should devise additional experiments focused on establishing the importance of endogenous IL-33 (rather than administered IL-33) on this immune response. This will help to establish the in vivo relevance of the work.

As stated above responding to the Reviewer #1 summary we (i) demonstrated release of endogenous IL-33 during *S. ratti* infection in lung and intestine explants (new Supplementary Figure S1) and (ii) analyzed mast cell activation in addition to parasite burden in *S. ratti*-infected mice where we suppressed (HpARI) or stabilized (CCP1/2) endogenous IL-33 that was naturally produced during infection (new Figure 3BC). These results show that both mast cell activation and parasite ejection are promoted by IL-33 stabilization *in vivo* and impaired by IL-33 suppression. We argue that this demonstrates the *in vivo* relevance of our follow up studies using rec IL-33 administration that triggered the same key mechanisms of intestinal parasite control.

Reviewer #2: The study is great and certainly worthy of publication. However a major weakness of the study which the authors need to address is to show endogenous IL-33 production following S. ratti infection.
The study as it stands very elegantly shows that addition of an exogenous source of IL-33 drives ILC2-IL-9 dependent mast cells responses.
The authors could set the scene for the study by showing that S. ratti induces a presumably epithelial IL-33 response. This could be demonstrated by ELISA and/or histologically (RNAScope gives good resolution).
Consideration should be made to including IHC of mast cell responses throughout the manuscript to help identify changes in location as well as magnitude of mast cell responses. Is the effect of IL-9/anti-IL-33 to reduce mast cell numbers or to impair mast cell responses. cKIT staining or similar could help to address this.

We have included data demonstrating that endogenous IL-33 is released by lung and intestine tissue explants during *S. ratti* infection (new Supplementary Figure S1), as suggested (page 5, lines 4ff). However, we would like to point out that we consider the exact quantification of the endogenous IL-33 that is released during infection to be difficult if not impossible, due the transient nature of IL-33 release *in vivo* (5) and the uncertainty surrounding the exact moment and site of damage-induced IL-33 release (dependent on parasite migration) in this model. As the “first wave” of IL-33 would be due to release of pre-formed nuclear IL-33 rather than de novo transcription and translation, quantification of IL-33 mRNA would indicate secondary responses (i.e. IL-33-inducing IL-33 (6) rather than primary release. The measurement of IL-33 protein from cultured tissue explants that we now show demonstrates increased release of the alarmin cytokine in infected vs naïve mice as such (new Supplementary Figure S1). Regarding the availability and subsequent function of this IL-33 *in vivo*, we feel that using inhibitors (HpARI) and stabilizers (CCP1/2) to IL-33 is addressing this point more directly. Our previous studies clearly showed that HpARI and CCP1/2 antagonized or stabilized IL-33 in a highly specific manner (1, 2). Therefore any *in vivo* effects of these proteins indicate (i) the presence of endogenous IL-33 and (ii) a non-redundant function of this IL-33 in control of intestinal parasite burden. In addition to the parasite burden, we now have evaluated mast cell responses in HpARI and CCP1/2 treated mice by measuring mMCPT-1 in the serum. These results are shown in the new Figure 3BC and demonstrate that endogenous IL-33 promotes mast cell activation which is the key mechanism of IL-33-induced intestinal anti-*S. ratti* immunity described for the treatment with exogenous IL-33. (page 7, lines 16 ff and page 13, lines 6 ff).

We agree that our data showing elevated mMCPT-1 levels does not discriminate between increased mast activation, increased mast cell numbers, or both. We have stated as such in the revised version of the manuscript (page 6, lines 31 ff and page 7 lines 1 ff). To harvest intestinal cells or prepare sections for histology we would need to perform a new set of infection experiments in HpARI and/or CCP1/2 treated and *S. ratti*-infected mice. This would delay re-submission of the manuscript by 4-5 month including the time needed to apply for permission for the extra animal experimentation as required under German law. However, as mMCPT-1 release is specific for activated mucosal mast cells (7) our results indicate that mast cell responses are critical in IL-33-mediated ejection of *S. ratti.*

Reviewer #3: None

Part III – Minor Issues: Editorial and Data Presentation Modifications
Please use this section for editorial suggestions as well as relatively minor modifications of existing data that would enhance clarity.

Reviewer #1: These were stated in Part I.

In addition, the second sentence of the introduction: "Taking advantage of the H. polygyrus-derived alarmin release inhibitor (HpARI)(29), we tested the impact of IL-33 on the early immune response to S. ratti as a model for any parasitic intestinal nematode with tissue-migrating life stages." seems overstated. I suggest removing the part of the sentence after "S. ratti".
We removed the second part of the sentence.

Reviewer #2: N/A

Reviewer #3: 1. It is suggested that the terms IL-33, mast cells and Strongyloides ratti be added to the keywords.
Done

2. Figure 1. There is large range in the results presented especially animals treated with HpARI in Panel B. It is stated that the data comes from 3 separate experiments. Did each of the 3 experiments have the same result, a wide range of responses? Did each individual experiment achieve statistical significance?

It is true that helminth infection models display a large inter- and intra-experimental variation in general. Regarding this experiments all 3 repeats show the same trend. If the experiments are analyzed separately it is not possible (due to sample number below 8) to perform an accurate normality test. Thus, non-gaussian distribution has to be assumed. According to a non-parametric test all three experiments do not show significant differences between PBS and HpARI. Once the results are pooled the normality test revealed a Gaussian distribution and the proper parametric analysis (ANOVA) revealed the significant difference indicated in the final figure.

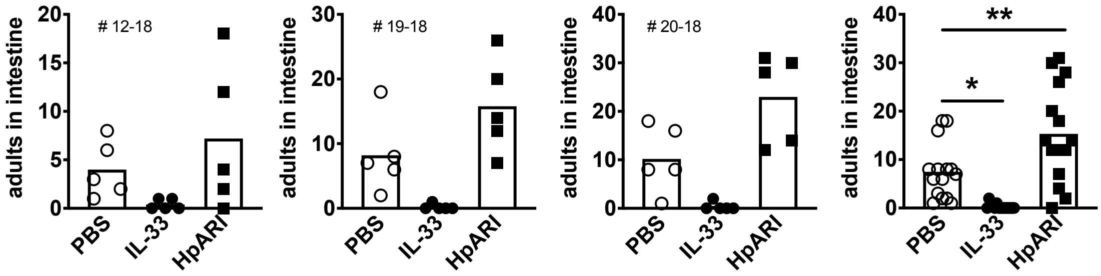

Figure 2: shown are the parasite burden recorded in the 3 individual experiments Exp # 12-18, 19-18 and 20 -18 leading to the final Figure 1B.

The figure legend does not explain the experiments presented in panel C. Corrected.

3. The legend for Figure 2 does not provide the needed information to interpret the figure.
We have added more detailed explanation regarding experiment design.

4. The legend in Figure 4 describing the open and closed circles and squares is placed in an obscure spot.
We changed the location of the legend.

5. It is unclear what the value of the data is on page 13 lines 1-15 and the authors should either explain what it contributes to the study or eliminate this section.

We agree that the incomplete ILC-depletion in SCID mice represents more an important “note of caution” that should be distributed among scientists during congresses but does not contribute to the specific story of the manuscript. Thus, we deleted this dataset and its discussion.

6. The authors should consider adding a figure illustrating the proposed sequence of events described on page 17 lines 6-10.
We have added a diagram as new Figure 6 describing our working hypothesis as suggested (page 12, lines 22 ff)

7. The paper would benefit from a concluding paragraph in which the authors provide context for the results from this study and how this new information might be applied to other infections or potentially to the development of new approaches to control infection. We have added a concluding paragraph (page 13 lines 6 ff).

In summary we feel that the revised version of the study addressed all points raised, thereby improving the manuscript. We would like to thank the reviewers again for their intellectual input and time and hope that they will be satisfied with our rebuttal and resubmission.

On behalf of the authors,
sincerely

Minka Breloer

---

literature

1. Osbourn M, Soares DC, Vacca F, Cohen ES, Scott IC, Gregory WF, et al. HpARI Protein Secreted by a Helminth Parasite Suppresses Interleukin-33. Immunity. 2017;47(4):739-51 e5.
2. Chauche C, Vacca F, Chia SL, Richards J, Gregory WF, Ogunkanbi A, et al. A Truncated Form of HpARI Stabilizes IL-33, Amplifying Responses to the Cytokine. Front Immunol. 2020;11:1363.
3. Blankenhaus B, Reitz M, Brenz Y, Eschbach ML, Hartmann W, Haben I, et al. Foxp3(+) regulatory T cells delay expulsion of intestinal nematodes by suppression of IL-9-driven mast cell activation in BALB/c but not in C57BL/6 mice. PLoS Pathog. 2014;10(2):e1003913.
4. Yasuda K, Muto T, Kawagoe T, Matsumoto M, Sasaki Y, Matsushita K, et al. Contribution of IL-33-activated type II innate lymphoid cells to pulmonary eosinophilia in intestinal nematode-infected mice. Proc Natl Acad Sci U S A. 2012;109(9):3451-6.
5. Cohen ES, Scott IC, Majithiya JB, Rapley L, Kemp BP, England E, et al. Oxidation of the alarmin IL-33 regulates ST2-dependent inflammation. Nat Commun. 2015;6:8327.
6. Hardman CS, Panova V, McKenzie AN. IL-33 citrine reporter mice reveal the temporal and spatial expression of IL-33 during allergic lung inflammation. Eur J Immunol. 2013;43(2):488-98.
7. Reynolds DS, Stevens RL, Lane WS, Carr MH, Austen KF, Serafin WE. Different mouse mast cell populations express various combinations of at least six distinct mast cell serine proteases. Proc Natl Acad Sci U S A. 1990;87(8):3230-4.
